# Loss of Sialic Acid Binding Domain Redirects Protein σ1 to Enhance M Cell-Directed Vaccination

**DOI:** 10.1371/journal.pone.0036182

**Published:** 2012-04-30

**Authors:** Dagmara Zlotkowska, Massimo Maddaloni, Carol Riccardi, Nancy Walters, Kathryn Holderness, Gayle Callis, Agnieszka Rynda-Apple, David W. Pascual

**Affiliations:** 1 Department of Food Chemistry, Institute of Food Research, Polish Academy of Science, Olsztyn, Poland; 2 Department of Immunology and Infectious Diseases, Montana State University, Bozeman, Montana, United States of America; National Council of Sciences (CONICET), Argentina

## Abstract

Ovalbumin (OVA) genetically fused to protein sigma 1 (pσ1) results in tolerance to both OVA and pσ1. Pσ1 binds in a multi-step fashion, involving both protein- and carbohydrate-based receptors. To assess the relative pσ1 components responsible for inducing tolerance and the importance of its sialic binding domain (SABD) for immunization, modified OVA-pσ1, termed OVA-pσ1(short), was deleted of its SABD, but with its M cell targeting moiety intact, and was found to be immunostimulatory and enhanced CD4^+^ and CD8^+^ T cell proliferation. When used to nasally immunize mice given with and without cholera toxin (CT) adjuvant, elevated SIgA and serum IgG responses were induced, and OVA-pσ1(s) was more efficient for immunization than native OVA+CT. The immune antibodies (Abs) were derived from elevated Ab-forming cells in the upper respiratory tissues and submaxillary glands and were supported by mixed Th cell responses. Thus, these studies show that pσ1(s) can be fused to vaccines to effectively elicit improved SIgA responses.

## Introduction

A number of strategies have been developed to improve parenteral and mucosal vaccine uptake, particularly those that adapt adhesins. Original strategies involved the coupling of vaccines to anti-Ig antibodies (Abs) [1] or MHC class II molecules [2] to develop an adjuvant-free method to enhance vaccine uptake. More recently, using Abs to target dendritic cells (DCs), as with DEC205 mAb, has enhanced immunogenicity to HIV [3] and plague LcrV [4]. Likewise, using a mAb to C-type lectin DC-specific intracellular adhesion molecule 3-grabbing nonintegrin has been used to stimulate human DCs in vitro from volunteers previously immunized with KLH-pulsed DCs [5]. Alternatively, using poly-γ-glutamic acid-based nanoparticles has also been used to effectively target DCs' phagocytic properties following parenteral immunization to stimulate proinflammatory responses that could ultimately protect against *Listeria monocytogenes* challenge [6]. Even small peptides, as shown using a 12-mer peptide, have been found to bind human DCs and when fused to *Bacillus anthracis* protective antigen, it facilitates antigen (Ag) uptake more effectively than without the DC-targeting peptide for conferring protection against *B. anthracis* Sterne challenge [7].

Both the described Ab- [1]–[5] and DC-targeting methods [3]–[7] adopt a targeting strategy for a specific host cell receptor. To improve mucosal immunity, strategies also have focused on targeting microfold (M) cells [8], a specialized epithelium present on the luminal surface of the Peyer's patches (PPs) or nasal-associated lymphoid tissue (NALT). M cells can sample luminal Ags or, in some cases, are targeted by intestinal pathogens [9], [10]. Consequently, M cells have been targeted to facilitate uptake of DNA vaccines using M cell ligands [11]–[13], including reovirus protein sigma one (pσ1) [11], [12], an anti-M cell mAb [14], or the B subunit for cholera toxin (CT-B) [15]–[19].

Because of its ability to bind to tissue M cells [20]–[24], we questioned whether pσ1could be adapted to deliver soluble Ags to the mucosa. Pσ1 is a highly structured protein composed of an elongated fibrous tail (T), which inserts into the virion, and a globular head (H), which protrudes from the virion to bind host cells. Pσ1 is subdivided into five distinct morphologic domains: T1 to T4 and H. The T1 domain, ∼25 residues long, forms an α-helical coiled-coil and turn; T2, ∼150 residues long, forms an α-helical coiled-coil, referred to as the shaft; T3, ∼65 residues, is an eight stranded β-sheet containing a sialic acid binding domain (SABD); and T4, ∼75 residues, contains the trimerizing domain and consists of a β-sheet structure flanked by two short regions of α-helical coiled-coil [21]. Finally, the H domain or head, ∼145 residues, is believed to assume a globular structure, as evidenced by electron microscopy and by its crystal structure [22]. Previous work has elegantly demonstrated that pσ1 mediates a multi-step adhesion process involving both protein- and carbohydrate-based receptors [23]. At least one cell receptor has been identified as the junction adhesion molecule 1 (JAM-1) [24].

**Figure 1:**
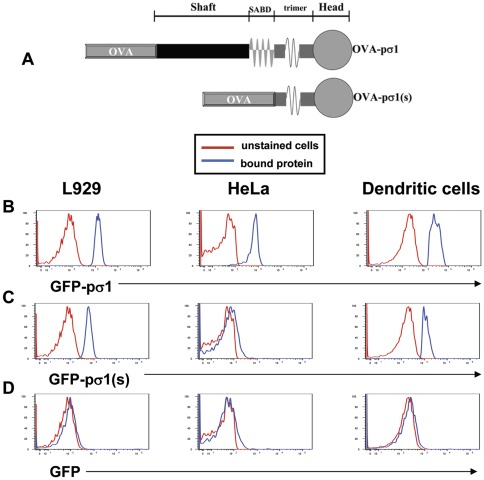
Deletion of pσ1's sialic acid binding domain (SABD) eliminates HeLa cell binding capacity. **A.** Schematic representation of OVA-pσ1 variants: tolerogenic OVA-pσ1 (shaft, SABD, trimerization domain [trimer], and head) and OVA-pσ1(short) [OVA-pσ1(s)] lacking the SABD and shaft. Cell binding to L929 cells, HeLa cells, and dendritic cells by recombinant **B.** GFP-pσ1, **C.** GFP-pσ1(s), and **D.** GFP are shown. GFP-pσ1(s) bound to L929 cells and to mouse DCs, but lost capacity to bind HeLa cells (sialic acid-dependent).

Our recent studies using soluble proteins genetically fused to pσ1 have proven successful in eliciting tolerance to OVA [25]–[27], proteolipid protein peptide [27], and myelin oligodendrocyte glycoprotein (MOG; [28], [29]). In fact, nasal or oral MOG-pσ1 treatment could reverse experimental autoimmune encephalomyelitis within 24 h of intervention [28], [29]. Pσ1 is self-tolerizing, allowing multiple doses if required [25], and it tolerizes passenger tolerogens, even in the presence of potent adjuvants CT and CpG [25]. Because of pσ1's capacity to induce tolerance to physically coupled proteins, we queried the relevance of pσ1's SABD to tolerance and immunity. Previous studies have established the importance of pσ1's H to L cell [30], [31] and M cell binding [11], [20], [26], [32], and thus, a mutant pσ1 was generated, lacking its SABD, but still retaining its H to maintain its targeting capabilities (Figure 1A). As a result, when ovalbumin (OVA) was genetically coupled to mutant pσ1, termed OVA-pσ1(short) [OVA-pσ1(s)], the ability to induce tolerance to OVA was abrogated. Instead, immunization occurred, and in fact, in the presence of co-administered adjuvant, OVA-pσ1(s) was 5-fold more effective per molar basis in stimulating anti-OVA Ab titers than native OVA. These results suggest that retention of pσ1's H was sufficient to improve OVA's immunogenicity.

## Results

### Differential cell binding activity in the absence of Pσ1's SABD

Previous work with OVA-pσ1 demonstrates it could induce tolerance to OVA when given nasally [25] or orally [26], and M cell uptake of OVA-pσ1 contributes to this tolerogenic response [25], [26]. Since others have shown that reovirus binding to host cells is a two-step process via pσ1's SABD and H [23], we queried whether pσ1's ability to stimulate tolerance is in part contributed to sialic acid binding. Thus, we hypothesized pσ1 could be further manipulated to direct an inflammatory rather than a tolerogenic response and still retain its ability to direct vaccines via its globular H to mucosal inductive tissues via M cells. To enable vaccination, pσ1 was truncated to eliminate its shaft and SABD, leaving the trimerization domain and head intact (Figure 1A) to allow the latter to mediate M cell binding [20]; the new truncated construct was termed pσ1(short) [pσ1(s)] (Figure 1A). To demonstrate the cell binding properties of pσ1(s), GFP-pσ1 and GFP-pσ1(s) were generated to facilitate detection by flow cytometry. As previously shown [23], [30], [31], intact pσ1 bound to both L and HeLa cells (Figure 1B). While still retaining its ability to bind to L cells, GFP-pσ1(s) lost its ability to bind to HeLa cells (Figure 1C). Intact GFP-pσ1 bound to DCs, but GFP-pσ1(s) showed reduced binding capacity to DCs, suggesting a portion of its binding interactions is sialic acid binding-dependent (Figure 1D). Collectively, these results demonstrate that pσ1(s) can retain its ability to bind to L cells via its H domain, but not to HeLa cells since its SABD is absent.

### Pσ1(s) retains ability to bind M cells

**Figure 2:**
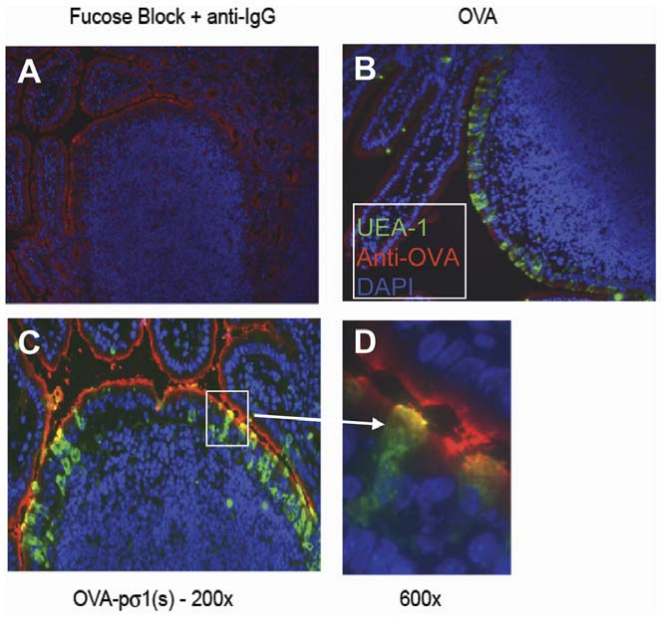
OVA-pσ1(s) retains the ability to bind PP M cells. An ileal loop assay was performed to assess the ability of OVA-pσ1(s) and OVA to bind to PP M cells as determined by co-localization with FITC-UEA-1. **A.** Fucose block of UEA-1 binding in the presence of biotinylated rabbit IgG and Streptavidin-Alexa® Fluor 594. **B.** OVA does not co-localize to PP M cells since no OVA could be detected with biotinylated rabbit anti-OVA plus Streptavidin-Alexa® Fluor 594. **C.** and **D.** OVA-pσ1(s) co-localizes with UEA-1 in PP M cells as detected with biotinylated rabbit anti-OVA plus Streptavidin-Alexa® Fluor 594 at **C.** 200× and **D.** 600×.

We have previously shown intact pσ1 is capable of binding to host PP [26] and NALT M cells [11]. The L cell binding data suggest pσ1(s) binding is primarily mediated via the H and is less dependent upon the SABD. To determine if OVA-pσ1(s) retains the ability to bind to M cells, an ileal loop assay was performed. Segments of small intestine were incubated with OVA or OVA-pσ1(s) for 1 hr and subsequently evaluated by immunofluorescence for their ability to bind to PP M cells (Figure 2). Using an anti-OVA Ab, OVA-pσ1(s) co-localized to PP M cells with FITC-UEA-1 (Figure 2C, D) similar to that seen with intact pσ1 [26], but in mouse intestinal loops incubated with OVA, no co-localization with FITC-UEA-1 was observed (Figure 2B). Thus, these data show OVA-pσ1(s) can still target host M cells in the absence of its SABD, suggesting vaccines can still be delivered to mucosal inductive tissues.

### Absence of SABD by Pσ1(s) stimulates, rather than inhibits, CD4^+^ and CD8^+^ T cells to OVA

**Figure 3:**
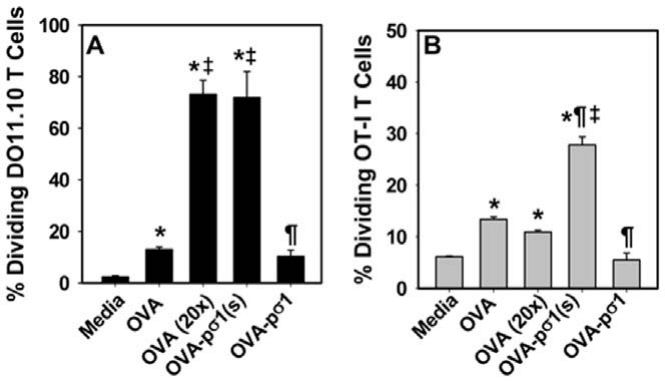
OVA-pσ1(s) is immunostimulatory and can induce transgenic DO11.10 CD4^+^ and OT-I CD8^+^ T cells to proliferate. Naive DCs were isolated and pulsed with media, 50 µg/ml, 1.0 mg/ml OVA (20×), 50 µg/ml OVA-pσ1(s), or 50 µg/ml OVA-pσ1, for 24 hrs and co-cultured with carboxy-fluorescein diactetate succinimidyl ester (CFDA)-labeled, purified **A.** DO11.10 CD4^+^ or **B.** OT-I CD8^+^ T cells for 4 days. Extent of T cell proliferation was measured by flow cytometry. Depicted are the mean ± SEM of two - three experiments: * *P*<0.001 vs. media; ^¶^ *P*<0.001 vs. OVA (20×); ^‡^ *P*≤0.002 vs. OVA-pσ1.

To test if OVA-pσ1(s) was tolerogenic or immunostimulatory, transgenic DO11.10 CD4^+^ (Figure 3 A) and OT-1 CD8^+^ T cells (Figure 3B) were cultured in the presence of Ag-pulsed DCs. These DCs were given 50 µg/ml OVA-pσ1, 50 µg/ml OVA-pσ1(s), 50 µg/ml OVA, or 1.0 mg/ml OVA overnight, washed, and co-cultured with CFDA-labeled DO11.10 or CFDA-labeled OT-I T cells for 4 days. Both OVA and OVA-pσ1(s) induced DO11.10 CD4^+^ and OT-I T cell proliferation, whereas OVA-pσ1 did not (Figure 3), suggesting OVA-pσ1(s) is immunostimulatory.

### Nasal OVA-pσ1(s) stimulates elevated Ab responses in the presence or absence of adjuvant

**Figure 4:**
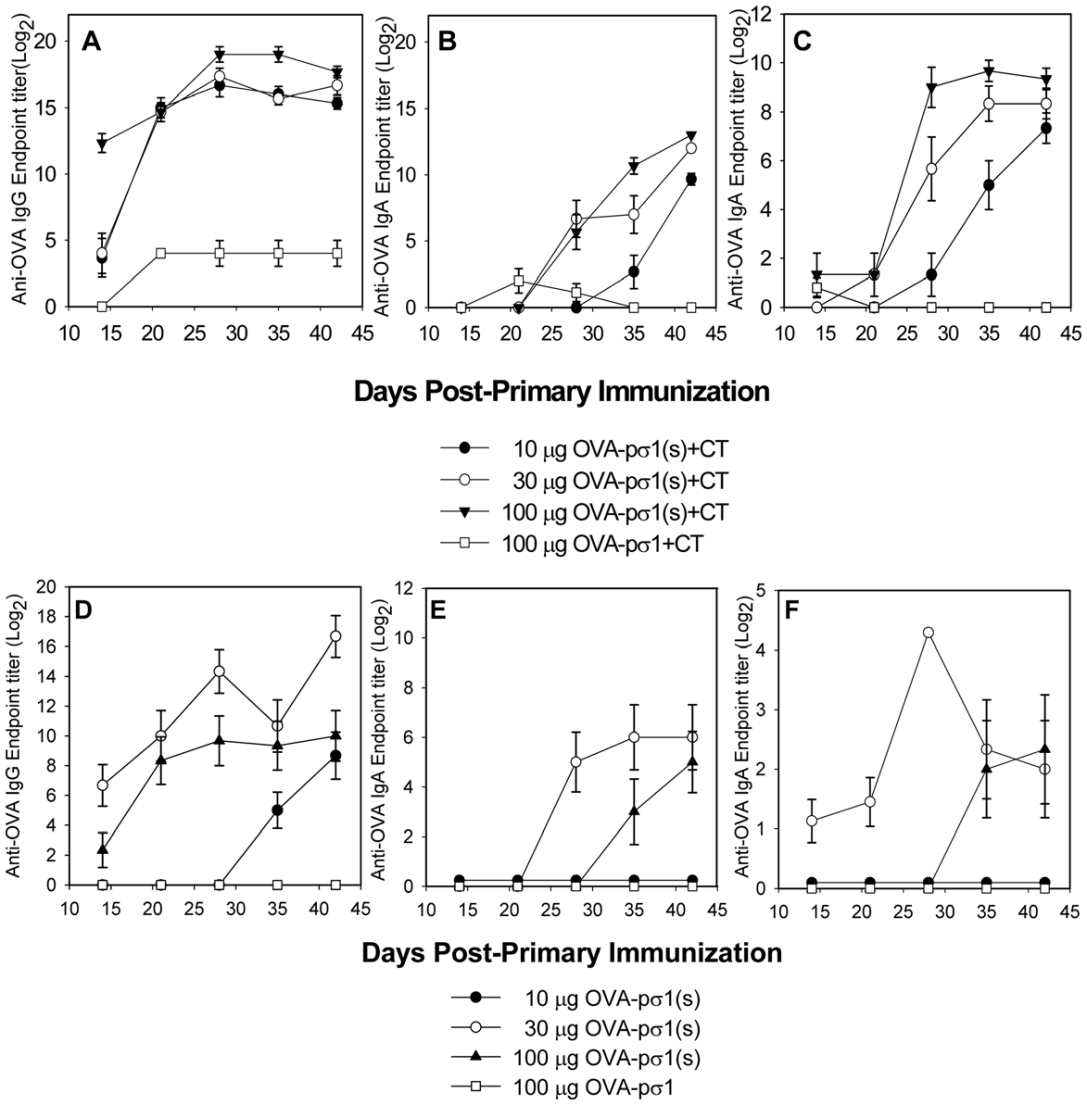
Nasal OVA-pσ1(s) stimulates Ab responses, unlike OVA-pσ1. C57BL/6 mice were nasally vaccinated with OVA-pσ1(s) or OVA-pσ1 plus **A.–C.** adjuvant or **D.–F.** without adjuvant, and OVA-specific **A.** and **D.** serum IgG, **B.** and **E.** serum IgA, and **C.** and **F.** fecal IgA Ab responses were measured. C57BL/6 mice (5/group) were dosed nasally with 10, 30, or 100 µg OVA-pσ1(s) or with 100 µg OVA-pσ1 with or without CT on days 0, 7, and 14. A kinetic analysis of the indicated time points is depicted as endpoint Ab titers from one of three experiments as mean ± SEM.

To learn OVA-pσ1(s)'s immunogenicity, a dose response and kinetic analysis were performed with groups of C57BL/6 mice nasally immunized with 10, 30, or 100 µg OVA-pσ1(s) with (Figure 4A–C) or without CT adjuvant (Figure 4D–F). Serum IgG anti-OVA end point titers were rapidly induced after the second (100 µg-dosed mice) or third immunization with OVA-pσ1(s) (30 µg-dosed mice), when given with CT, with an endpoint titer as great as 2^19±0.58^ (Figure 4A). Serum IgA anti-OVA titers were delayed for all groups and not detectable until after day 21, but these continued to increase to day 42 post-primary immunization (Figure 4B). In the absence of co-administered CT, serum IgG anti-OVA Abs were still greatly induced with the 30 µg OVA-pσ1(s) peaking at 2^16.7±1.39^ (Figure 4D), but showed a reduced serum IgA response although better than mice dosed with 10 µg or 100 µg (Figure 4E; P<0.05). Mucosal IgA anti-OVA Ab titers were potentiated in the presence of CT evidenced by the enhanced fecal IgA responses with either 30 or 100 µg dose (Figure 4C) when compared to mice immunized with 30 µg OVA-pσ1(s) alone with mucosal IgA Ab titers of 2^4.3^ (Figure 4F). In either the presence (Figure 4A–C) or absence of CT (Figure 4D–F), mice immunized with OVA-pσ1 failed to elicit appreciable serum IgG, IgA, or mucosal IgA anti-OVA responses consistent with our previous reports that OVA-pσ1 induces Ag-specific unresponsiveness [22], [23]. Thus, these results show OVA-pσ1(s) can induce both systemic and mucosal Ab responses to ferried OVA, unlike OVA-pσ1, and these responses are potentiated in the presence of co-administered adjuvant.

### Nasal OVA-pσ1(s) immunization lessens the amount of vaccine required for immunization

Similar to that done with OVA-pσ1(s), a dose and kinetic analysis was also performed to determine how effective 30 or 100 µg native OVA without or with CT performed (Figure S1). By 35 days post-primary immunization, mice immunized with 30 or 100 µg OVA, in the absence of CT, showed similar serum IgG Ab titers, as did mice nasally immunized with OVA plus CT (Figure S1A). Likewise, OVA plus CT-immunized showed no significant differences in serum IgA anti-OVA titers, and low serum IgA titers were obtained in mice immunized with OVA alone (Figure S1B). For SIgA responses, CT adjuvant was required and optimally induced by mice immunized with the 100 µg OVA plus CT (Figure S1C–D). Thus, given these findings, mice evaluated in subsequent experiments used the 100 µg OVA plus CT dose.

**Figure 5:**
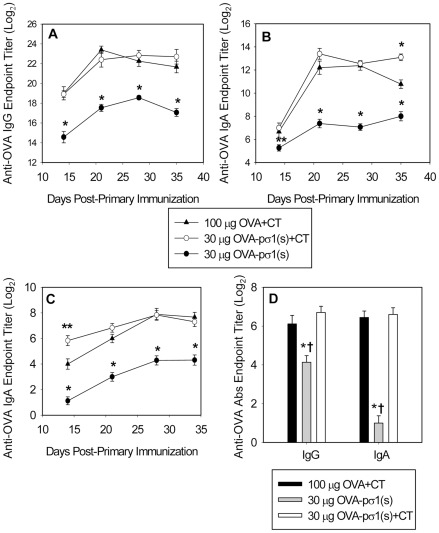
OVA-pσ1(s) is 5 times more effective per molar basis than native OVA in stimulating immune Abs. Groups of C57BL/6 mice (9/group) were immunized nasally with 30 µg OVA-pσ1(s) alone, 30 µg OVA-pσ1(s) plus cholera toxin (CT), or 100 µg OVA plus CT using the vaccination schedule described in Fig. 4. **A.–D.** Although mice immunized with OVA-pσ1(s) alone consistently produced less Ag-specific Abs, CT co-administration enhanced anti-OVA endpoint titers than the 100 µg OVA plus CT-immunized group: **A.** serum IgG, **B.** serum IgA, **C.** fecal IgA, and **D.** day 35 nasal wash IgA and IgG endpoint anti-OVA Ab titers. A kinetic analysis of the indicated time points is depicted from two experiments as mean ± SEM; *P<0.001, **P≤0.012 versus OVA plus CT-immunized mice; and ^†^ *P*<0.001 versus nasal wash titers from OVA-pσ1(s) plus CT-immunized mice.

Although the results obtained from mice immunized with a 30 or 100 µg OVA-pσ1(s) dose produced similar responses, it appeared the 30 µg dose was sufficient to stimulate a potent Ab response in the presence of CT. To test the effectiveness of OVA-pσ1(s) as an immunogen relative to OVA, groups of mice were nasally immunized with 30 µg OVA-pσ1(s) alone or with CT and another group with 100 µg OVA+CT (optimal dose of OVA). Both groups of mice adjuvanted with CT showed similar serum IgG anti-OVA Ab titers, and even mice immunized with OVA-pσ1(s) alone showed elevated Ab titers, but significantly less (P<0.001) than the adjuvanted groups (Figure 5A). Serum IgA Ab titers mimicked serum IgG responses, although OVA-pσ1(s) plus CT-dosed mice showed sustained IgA Ab titers when compared to OVA plus CT-dosed mice (P≤0.001; Figure 5B). Fecal IgA Ab titers for both the 30 µg OVA-pσ1(s) plus CT-dosed and 100 µg OVA plus CT-dosed mice were similar, and the addition of CT clearly potentiated the mucosal IgA response when compared to mice dosed with OVA-pσ1(s) alone (Figure 5C). Nasal wash data revealed no significant differences in IgG or IgA Abs among CT-adjuvanted groups, but these were significantly different (P<0.001) from mice given OVA-pσ1(s) alone (Figure 5D). Collectively, these studies demonstrate pσ1(s) directs mucosal and systemic Ab responses to OVA more efficiently by ∼5-fold on a molar basis than native OVA.

**Figure 6:**
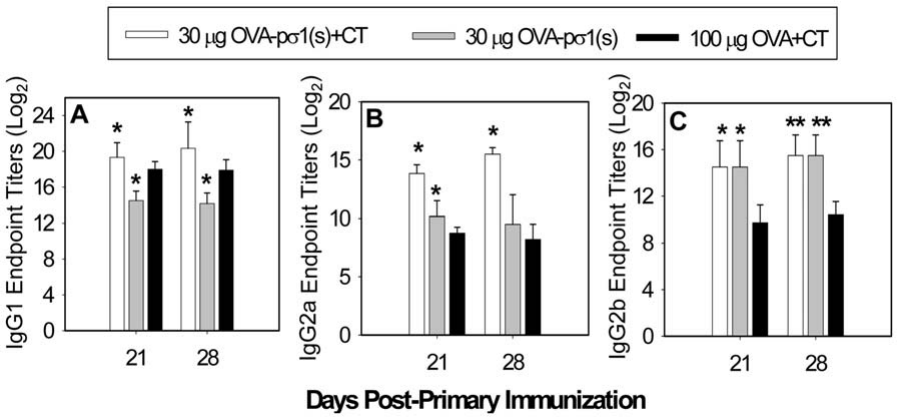
OVA-pσ1(s) plus CT stimulates greater IgG subclass Ab titers than OVA plus cholera toxin (CT). The same groups of immunized mice in Figure 5 were evaluated for their **A.** IgG1, **B.** IgG2a, and **C.** IgG2b subclass responses to OVA on days 21 and 28 post-primary vaccination. The indicated time points are depicted from two experiments as mean ± SEM; *P<0.001, **P<0.05 versus OVA plus CT-immunized mice.

IgG subclass responses induced in OVA-pσ1(s) plus CT-immunized mice relative to OVA plus CT-immunized mice were significantly enhanced (P<0.001) at both 21 and 28 days post-primary immunization. IgG1 titers were slightly, but significantly, elevated when compared to OVA plus CT-immunized mice (Figure 6A). Serum IgG2a anti-OVA Ab titers by OVA-pσ1(s) plus CT-immunized mice were elevated 34- and 158-fold on days 21 and 28, respectively, when compared to OVA plus CT-immunized mice (P<0.001; Figure 6B). Likewise, IgG2b anti-OVA Ab titers were significantly enhanced (P<0.001) by ∼30-fold versus OVA plus CT-immunized mice on days 21 and 28 (Figure 6C). Interestingly, even in the absence of adjuvant, OVA-pσ1(s)-immunized mice showed significantly greater IgG2a and IgG2b anti-OVA titers than OVA plus CT-immunized mice (Figure 6B, C). Mice immunized nasally with 30 or 100 µg OVA plus CT showed no differences in IgG subclass responses (Figure S2).

### OVA-pσ1(s) plus CT enhances mucosal inductive and effector tissue B cell responses

**Figure 7:**
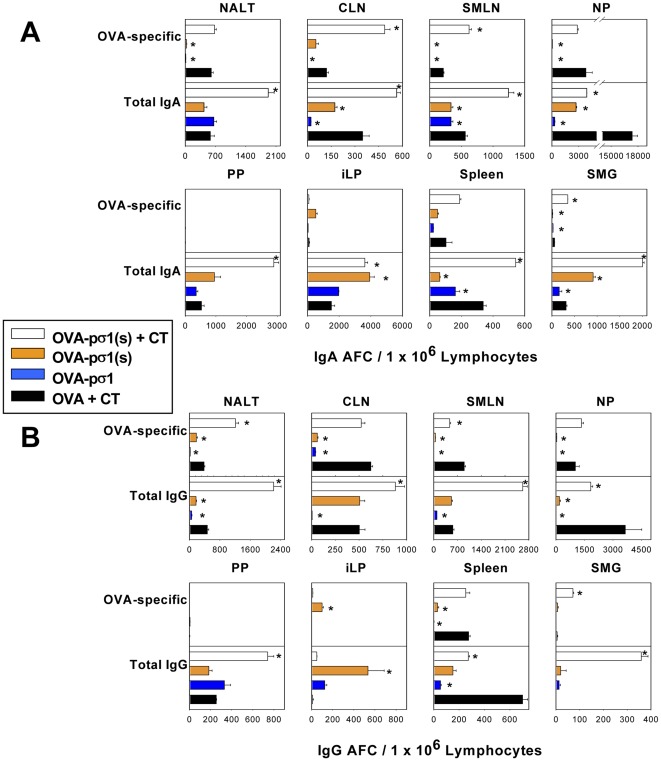
Nasal OVA-pσ1(s)+cholera toxin (CT) enhances A. IgA and B. IgG antibody-forming cell (AFC) responses. C57BL/6 mice were nasally immunized with OVA-pσ1(s) alone, OVA-pσ1alone, OVA-pσ1(s) plus CT, or OVA plus CT. OVA-specific and total **A.** IgA and **B.** IgG AFCs were measured by B cell ELISPOT conducted on day 21 post-primary immunization, and NALT, CLNs, SMLNs, nasal passages (NPs), PPs, small intestinal lamina propria (iLP), spleens, and SMGs were evaluated. OVA-pσ1(s) plus CT-immunized mice showed enhanced AFC responses in their CLNs, submaxillary LNs (SMLNs), and submaxillary glands (SMGs) when compared to OVA plus CT-immunized mice. Values are the mean ± SEM of AFC responses taken from two experiments, *P≤0.001, **P≤0.01 vs. OVA plus CT-immunized mice.

To establish the source of the induced B cells responsible for the enhanced mucosal IgA responses, mice were nasally immunized with 30 µg OVA-pσ1(s) or 100 µg OVA combined with CT; separate groups were given 30 µg OVA-pσ1(s) or OVA-pσ1 alone. Three wks after primary immunization, a B cell ELISPOT assay was performed to evaluate the source of OVA-specific IgA and IgG responses in the NALT, cervical lymph nodes (CLNs), submaxillary LNs (SMLNs), nasal passages (NPs), PPs, small intestinal lamina propria (iLPs), spleens, and submaxillary glands (SMGs) (Figure 7A, B). The OVA-pσ1(s) plus CT-immunized mice induced significantly greater (P≤0.001) IgA responses in the CLNs, SMLNs, and SMGs (Figure 7A), and IgG responses in the NALT and SMGs (Figure 7B) when compared to OVA plus CT-immunized mice. For head and neck tissues, OVA-pσ1(s) plus CT immunized mice showed more enhanced IgA and IgG AFC responses (P≤0.001) than mice immunized with OVA-pσ1. This lack of AFC responses by mice given OVA-pσ1 is consistent with our previous findings showing that intact pσ1 induces unresponsiveness, rather than active immunity, even when OVA-pσ1 is co-administered with potent adjuvants [25]. Thus, these studies show OVA-pσ1(s) in the absence of its SABD enhances Ag-specific B cell responses in both the mucosal and systemic compartments.

### OVA-Pσ1(s) plus CT induces a mixed Th cell response

**Figure 8:**
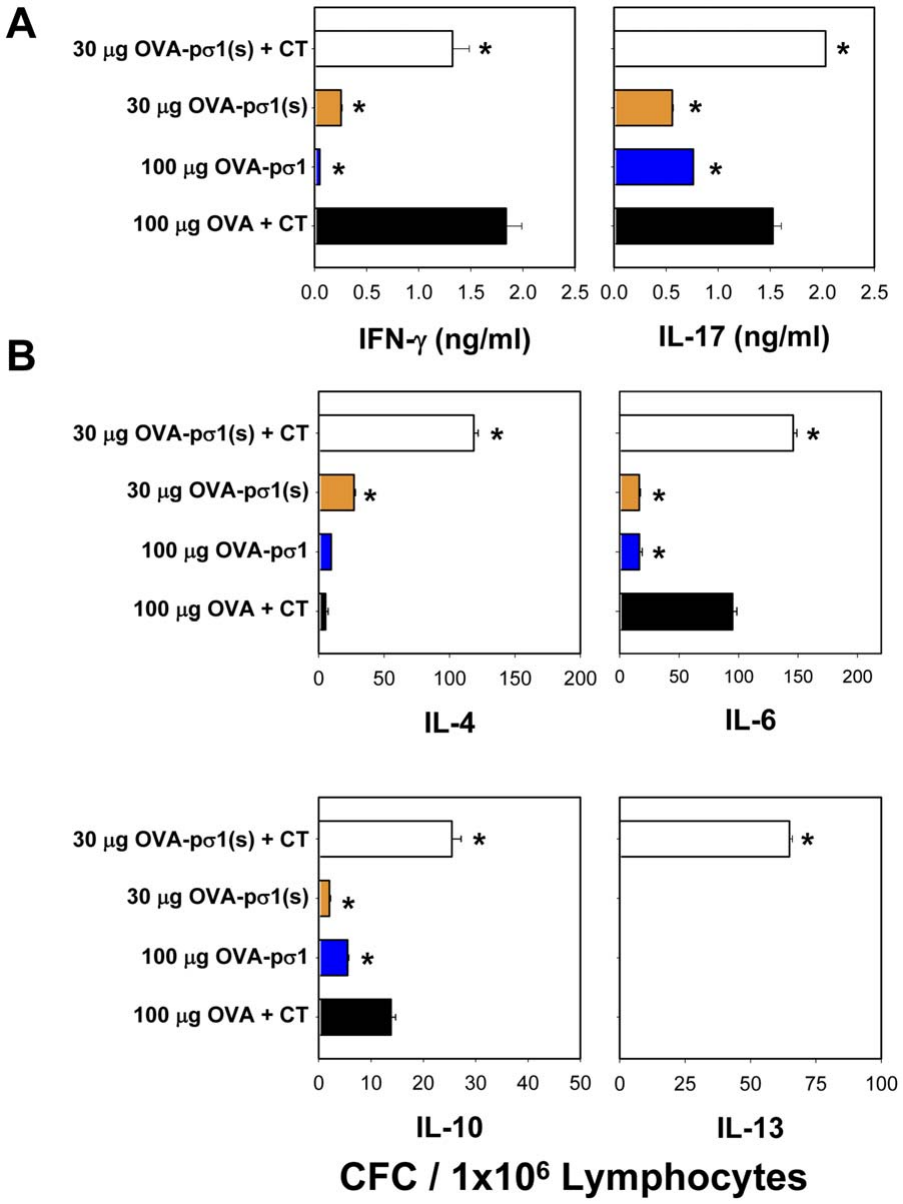
Nasal OVA-pσ1(s) stimulates mixed Th cell responses. OVA-pσ1(s) plus cholera toxin (CT) enhances splenic OVA-specific Th2 and Th17 cell responses when compared to mice nasally immunized with OVA-pσ1(s) alone, OVA-pσ1 alone, or with OVA plus CT-immunized mice. On day 24 post-primary immunization, CD4^+^ T cells were isolated and cultured in the presence of OVA or media for 2 days, and subsequently assessed for **A.** IFN-γ and IL-17 production by ELISA and **B.** IL-4, IL-6, IL-10, and IL-13 cytokine-forming cell (CFC) responses by T cell ELISPOT. Depicted are the means (corrected for media values) ± SEM of two experiments: * *P*<0.001 versus OVA plus CT-immunized mice.

To assess the CD4^+^ T cells supporting the observed elevated Ab responses, cytokine analysis was performed. IFN-γ and IL-17 secretion levels were similar between both groups of mice immunized with OVA-pσ1(s) plus CT and OVA plus CT (Figure 8A). However, CD4^+^ T cells from mice nasally immunized with 30 µg OVA-pσ1(s) plus CT showed significantly greater (P<0.001) IL-4, IL-6, IL10, and IL-13 CFC responses than mice nasally immunized with 100 µg OVA plus CT or from mice immunized with 30 µg OVA-pσ1(s) or 50 µg OVA-pσ1 alone (Figure 8B). Although Th2 cells are elevated, OVA-pσ1(s) plus CT immunization results in greater Th17 cell responses than mice immunized with OVA plus CT.

## Discussion

The mucosal immune system consists of the two functionally distinct types of tissues: 1) inductive sites where naive B and T cells come in contact with Ag; and 2) effector sites where activated B and T cells, after Ag priming in inductive sites, express their effector functions [31], [32]. Inductive sites are where Ag is first encountered and processed, and the initial induction of immune and memory B and T cells occurs. In the gut, the PPs represent inductive sites for the gut-associated lymphoreticular tissue (GALT) [33], [34], and similar lymphoid sites recently identified for the upper airways in rodents are referred to as NALT [35], [36]. These inductive tissues can be functionally and anatomically separated into three distinctive areas: 1) the dome with a unique lymphoepithelium; 2) the B cell follicles, usually containing one or more germinal centers; and 3) the perifollicular or T cell-dependent area. The dome region is covered by the epithelial layer termed follicle-associated epithelium (FAE), and within this epithelium are specialized epithelial cells or M cells that facilitate luminal Ag sampling [36]. Some intestinal pathogens can infect the host via M cells [9], [10], such as reovirus types 1 and 3 [20], [32] and *Salmonella* [37].

Given certain pathogens can exploit M cells for infection [9], [10], [20], [32], [37], and attenuated *Salmonella* vaccines are effective in stimulating mucosal and systemic immune responses [38], we questioned whether a soluble vaccine could be generated with the M cell targeting capabilities of reovirus and *Salmonella*. Initial work shows poly-L-lysine modification of pσ1 enables mucosal DNA vaccination [11], [12], and such modification does not inhibit its ability to bind to M cells [11]. Yet, a soluble fusion vaccine using OVA-pσ1 induces tolerance to both OVA and pσ1, even in the presence of potent adjuvants [25], suggesting poly-L-lysine modification of pσ1 may inadvertently interrupt or block its SABD's binding activity, making pσ1 immunogenic [25]. Herein this study, ablation of SABD clearly modified pσ1's immunogenicity and elevated mucosal and systemic Ab responses to the genetically fused Ag OVA subsequent nasal OVA-pσ1(s) administration. OVA-pσ1(s) was still found to bind to PP M cells in an ileal loop assay, unlike native OVA, which did not appear to bind to M cells. The cell binding by OVA-pσ1(s) to the PP M cells resembled our previous binding data for PP [26] and NALT [11], suggesting it retained its ability to target mucosal inductive tissues. Thus, in the absence of its SABD, OVA-pσ1(s) retained its M cell binding capacity despite losing its ability to bind to HeLa cells.

To test the influence of its SABD upon tolerance or immunogenicity, experiments were conducted to measure induced Ab responses to OVA when OVA-pσ1(s) was used to immunize mucosally in the absence or presence of co-administered adjuvant. To determine if OVA-pσ1(s) alone could induce significant anti-OVA Ab responses without adjuvant, three 30 µg doses of OVA-pσ1(s) at weekly intervals were sufficiently effective to elicit elevated serum IgG anti-OVA Ab levels. In fact, even after two doses, these were markedly enhanced; however, fecal or nasal IgA Abs levels were not greatly induced, suggesting that in the absence of adjuvant the pσ1(s) vaccine platform may be effective if the desired endpoint only requires a systemic Ab response. Mice given 100 µg OVA alone produced serum IgG titers resembling mice vaccinated with 10 µg OVA-pσ1(s), and no mucosal Ab responses could be detected (data not shown). Co-administration of CT with OVA-pσ1(s) amplified both the serum IgG and mucosal IgA anti-OVA Ab responses significantly more than mice immunized with OVA-pσ1(s) alone. Importantly, when compared to native OVA plus CT immunization, on a per molar basis, OVA-pσ1(s) required 5-fold less OVA than the 100 µg dose of OVA used to stimulate equivalent systemic and mucosal Ab responses. While serum IgG responses were similar in mice nasally immunized with 30 or 100 µg OVA, their fecal IgA responses did differ for the 100 µg-dosed mice, showing prolonged and greater SIgA anti-OVA titers. At the 100 µg OVA dose, fecal and nasal wash IgA anti-OVA Ab responses by mice immunized, either with native OVA plus CT or OVA-pσ1(s) plus CT, exhibited similar mucosal IgA responses, and these were of similar magnitude obtained with mice nasally immunized with OVA plus CT [25]. Thus, as demonstrated here, vaccine targeting can enhance and can certainly induce more rapid systemic and mucosal Ab responses.

Evaluation of the supportive Th cells for the OVA-specific Ab responses revealed modest Th2-type production when OVA-pσ1(s) plus CT was used to immunize mice. Both IFN-γ and IL-17 were markedly induced by OVA-pσ1(s) plus CT-immunized mice when compared to OVA plus CT-immunized mice. It was recently shown that nasal CT immunization can stimulate IL-6- and IL-17-producing CD4^+^ T cells [39], [40], and our results confirmed this finding. In this regard, OVA-pσ1(s) plus CT-immunized mice showed elevated Th17 cell responses significantly greater than OVA plus CT-immunized mice and provided a method for potentially vaccinating against pathogens requiring Th17 cells to resolve infections, such as with *Streptococcus pneumoniae* [41] or *Pseudomonas aeruginosa* [42]. Although a mixed Th cell response was induced, these data show OVA-pσ1(s) in the presence of CT can elicit an elevated Th17 cell response.

To begin to understand the mechanism of action by pσ1(s), additional analyses were performed to assess how OVA-pσ1(s) can be immunogenic. Priming DCs with OVA-pσ1(s) was more efficient than with native OVA since usually 1.0 mg/ml was required for native OVA [43] rather than the 50 µg/ml OVA-pσ1(s) used in this study, and when this lesser dose (50 µg/ml) of native OVA was used, suboptimal priming was obtained. This in vitro finding further confirmed our in vivo results showing the improved efficiency of using OVA-pσ1(s). Unlike intact OVA-pσ1, which failed to induce a proliferative response, OVA-pσ1(s) could stimulate DO11.10 CD4^+^ T and OT-I CD8^+^ T cells to proliferate, unlike OVA-pσ1, as previously demonstrated for CD4^+^ T cells [25], [26]. OVA-pσ1 also failed to stimulate CD8^+^ T cells, a finding not previously described. In fact, OVA-pσ1 was found to be tolerogenic and stimulated the production of IL-10 [25]–[29] or TGF-β [26], [27] and suppressed IFN-γ and IL-17 [25]–[28]. Since OVA-pσ1(s) lacked the SABD, an immunostimulatory response was induced, suggesting the SABD also contributes to tolerance induction.

In conclusion, the collective data demonstrate OVA-pσ1(s) is immunostimulatory in the absence of binding sialic acids, unlike its parent molecule, OVA-pσ1, which contains its SABD and results in tolerance induction. In fact, pσ1-mediated tolerance has been observed with other Ags, including proteolipid protein peptide [27] and myelin oligodendrocyte glycoprotein [28], [29]. Consequently, OVA-pσ1(s) still retains its M cell targeting moiety to enable more effective mucosal vaccination than OVA alone. Such findings expand the pσ1 delivery platform for mucosal immunization.

## Methods

### Ethics statement

All animal care and procedures were in accordance with institutional policies for animal health and well-being and approved by Montana State University Institutional Animal Care and Use Committee under protocol 52.

### Construction and expression of OVA-pσ1(s)

OVA-pσ1(s) is a deletion mutant of OVA-pσ1 [25] encompassing the entire OVA gene fused to the last 207 amino acids of pσ1, leaving a minimal binding subunit that includes the trimerizing domain and H (Figure 1A). OVA was amplified with a 5′ primer featuring an EcoR1 site and an ATG codon and a 3′ Sal1 primer. The upstream primer for pσ1 contained a SalI site designed to frame the OVA; the downstream primer contained a KpnI primer designed to frame the fused protein to the His-tag present in the *Pichia pastoris* expression vector pPICB. The PCR products were gel-purified and cloned into a topocloning vector. The inserts were then excised by cutting with the appropriate pairs of restriction enzymes (New England Labs, Beverly, MA) and gel-purified again. Finally, the yeast expression vector was cut with EcoRI and KpnI. A tripartite ligation was set up to ligate: 1) the “passenger Ag" (OVA), as an EcoRI-SalI fragment; 2) the “transporter," as a *Sal*I-*Kpn*I fragment; and 3) the vector cut with *Eco*RI and *Kpn*I. The junction between the “passenger Ag" and the “transporter" featured a flexible linker (Gly-Arg-Pro-Gly) to minimize steric hindrance between the components. To construct GFP-pσ1, the gene encoding for pσ1 was amplified with a 5′ EcoRI primer and a 3′ SalI primer, gel-purified, cloned into *E.coli* via a topocloning vector, excised with EcoRI-SalI, repurified, and cloned into the *P. pastoris* pPICZA expression vector cut with EcoRI-SalI. The resulting construct was modified by inserting an EcoRI fragment containing a GFP gene that was amplified from pLANTERN (Gibco-BRL) as a template. Primers were designed to frame GFP into pσ1 and the fusion into the His-tag of the vector. To construct GFP-pσ1(s), the same strategy was followed, but only the appropriate deletion primer was designed to amplify the last 207 amino acids of the pσ1. The resulting constructs were sequenced and expressed in the yeast *P. pastoris*, according to the manufacturer's directions (Invitrogen Corp., Carlsbad, CA). Recombinant proteins were extracted from yeast cells by a bead-beater (Biospec Products, Bertlesville, OK) and purified on a Talon metal affinity resin (BD Biosciences, Palo Alto, CA), according to manufacturer's instructions. Proteins were assessed for purity and quality by Coomassie-stained polyacrylamide gel electrophoresis. OVA-pσ1(s) migrated as a single band with the expected MW ∼68,300 Da.

### Cell binding assays

L929 cells (ATCC, CCL-1, Manassas, VA) and HeLa cells (ATCC, CCL-2) were grown in a complete medium (CM): RPMI medium supplemented with FBS (Atlanta Biologicals, Atlanta, GA), 1 mM nonessential amino acids, 1 mM sodium pyruvate, 1 mM HEPES, 100 µg/ml penicillin, and 100 µg/ml streptomycin. DCs were enriched from head and neck lymph nodes and spleens, similar to that previously described [44]. Briefly, tissues were subjected to collagenase (50 U/ml Type IV; Sigma-Aldrich, St. Louis, MO) digestion+DNAse (0.8 U/ml; Promega, Madison, WI) in teflon flasks with gentle stirring for 30 min at 37°C. The digested tissues were passed through Nitex (Fairview Fabrics, Hercules, CA) and incubated in CM at 37°C for 30 min. Cell suspensions were washed in CM and then subjected to density gradient centrifugation using a modified technique [44]. Total lymphocytes were resuspended in 2.0 ml of Hank's Balanced Salt Solution (HBSS; GIBCO-Invitrogen Corp.) and then added to 1.0 ml Optiprep™ (Axis-Shield PoC AS, Oslo, Norway) and mixed gently. Cells were then layered onto the density gradient with a 1∶3.2 (14.3%) solution of diluent, which consisted of 0.88% NaCl, 1.0 mM EDTA, and 0.5% (w/v) BSA, and 10 mM Hepes-NaOH, pH 7.4) and Optiprep™. The gradient was then topped with 3.0 ml of HBSS. Lymphocytes were subjected to density gradient centrifugation for 15 min at 20°C. DCs were removed from the top of the Optiprep gradient and washed in CM. Typically, DCs were enriched to >85% purity, as evaluated by immunofluorescent staining with anti-CD11c (B–D Pharmingen, San Diego, CA) and anti-CD205 (DEC205; Serotec, Inc., Raleigh, NC) mAbs.

L and HeLa cells (3×10^4^) and DCs (3×10^5^) were resuspended in 200 µl of FACS buffer (Dulbecco PBS+2% FBS) containing equimolar GFP concentration relative to 10 µg of GFP-pσ1: 7.5 µg of GFP-pσ1(s) and 3.9 µg GFP. These were incubated on ice for 20 min. Cells were washed once with 3 ml of FACS buffer and resuspended in FACS buffer and subjected to FACS analysis on a FACSCalibur flow cytometer (BD Biosciences).

### Immunofluorescent detection of OVA-pσ1(s) binding to M cells

An ileal method was used to detect OVA-pσ1(s) binding to PP M cells similar to that previously described [45]. Ileal loops were injected with 200 µg OVA-pσ1(s) or OVA and incubated for 1 hr; ileal loops were removed and injected with OCT® cryoembedding media (Sakura Finetek, Torrance CA) to distend the lumen. The OCT filled loop was embedded in OCT, snap frozen with dry ice, cooled in 2-methylbutane, and sectioned into 5 µm using a cryostat. Frozen sections were picked up on positive charge slides, air dried at room temperature overnight, fixed at room temperature in 75 ml acetone/25 ml absolute ethanol for 5 minutes, and rinsed immediately after fixation with 3 changes of rinse buffer (Dulbeccos PBS/0.025% Tween 20). Sections were protected from light during staining protocol. Sections were stained with 8 µg/ml *Ulex Europaeus agglutinin 1* conjugated to FITC (UEA-1-FITC; Vector Laboratories, Burlingame, CA) in rinse buffer for 30 min at room temperature. To block UEA-1 binding, 8 µg/ml UEA1-FITC in 300 mM L-fucose was incubated overnight at 4°C before applied to a section, as described above. Sections were rinsed and then blocked with 10% normal goat serum/1.5% mouse serum in rinse buffer for 30 min followed by Streptavidin/Biotin blocking kit (Vector Laboratories). A biotinylated rabbit anti-OVA Ab (5 µg/ml; Sigma-Aldrich) diluted in 10% goat/2.5% mouse serum in rinse buffer was applied to sections for 30 min at room temperature. The negative control used was 5 µg/ml biotinylated rabbit IgG in same diluent and served as primary Ab. Sections were rinsed, then incubated for 30 min at room temperature with 2 µg/ml Streptavidin-Alexa® Fluor 594 (Molecular Probes/Invitrogen, Eugene, OR) diluted in rinse buffer, and then rinsed and cover slipped with Prolong Gold antifade reagent with DAPI (Molecular Probes/Invitrogen).

### Assessment of OVA-pσ1(s)'s proliferative capacities

HNLN and splenic DCs were isolated, as described above, via Optiprep™ gradient centrifugation. The DCs were pulsed overnight with media, 50 µg/ml OVA-pσ1, 50 µg/ml OVA-pσ1(s), 50 µg/ml OVA, or 1.0 mg/ml OVA (grade V; Sigma-Aldrich) [25], [43], and the next day, DCs were washed in CM. DO11.10 CD4^+^ T cells were isolated by negative selection (Dynal Mouse CD4 Negative Isolation Kit, Invitrogen), as were OT-I CD8^+^ T cells (Dynal CD8 Negative Isolation Kit, Invitrogen), and each was labeled with 1.25 µM carboxy-fluorescein diactetate succinimidyl ester (CFDA; Molecular Probes-Invitrogen) in RPMI for 5 min at room temperature in the dark and then washed three times. The labeled DO11.10 CD4^+^ and OT-I CD8^+^ T cells were added to the Ag-pulsed DCs at 2∶1 ratio, and lymphocytes were cultured for 4 days. After culture, labeled DO11.10 CD4^+^ and OT-I CD8^+^ T cells were evaluated for extent of their proliferation by flow cytometry.

### Mice and immunizations

C57BL/6N and BALB/c mice (Frederick Cancer Research Facility, National Cancer Institute, Frederick, MD) and transgenic DO11.10 mice [25] were used throughout this study. All mice were maintained at Montana State University Animal Resources Center under pathogen-free conditions in individually ventilated cages under HEPA-filtered barrier conditions and fed sterile food and water ad libitum. The mice used were 6 to 8 wks of age and free of bacterial and viral pathogens, as determined by Ab screening and histopathologic analysis of major organs and tissues. All animal studies were approved by the MSU Institutional Animal Care and Use Committee.

C57BL/6 mice (5 per group) were nasally immunized with 10, 30, or 100 µg of OVA-pσ1(s), OVA-pσ1, or OVA (Sigma-Aldrich) without and with 2.0 µg CT adjuvant on days 0, 7, and 14. Blood (from saphenous vein) and mucosal samples were collected weekly beginning day 14. Fecal extractions and vaginal washes were performed, as previously described [25].

### Ab detection assays

Serum and fecal samples were evaluated for anti-OVA endpoint Ab titers by ELISA similar to that previously described [25], using purified OVA (Grade V, Sigma-Aldrich) as the coating Ag. Specific reactivity to OVA was determined using HRP conjugates of goat anti-mouse IgG-, IgG1-, IgG2a-, IgG2b-, and IgA-specific Abs (1.0 mg/ml; Southern Biotechnology Associates, Birmingham, AL), and ABTS (Moss Inc., Pasadena, CA) enzyme substrate. The absorbencies were measured at 415 nm on an ELx808 microplate reader (Bio-Tek Instruments). Endpoint titers were expressed as the reciprocal dilution of the last sample dilution, giving an absorbance of 0.1 OD units above the OD415 of negative controls after 1 h incubation.

To assess OVA-specific and total Ab-forming cell (AFC) responses, the B cell ELISPOT method was used [11], [13], [26]. Single cell suspensions were prepared from the CLNs, SMLNs, NALT, SMGs, NPs, iLP, PPs, and spleens of mice immunized with OVA plus CT, OVA-pσ1, and OVA-pσ1(s) with and without CT. Mucosal inductive tissues and spleens were isolated by conventional methods, similar to those previously described [11], [13], [25]. Briefly, dounce homogenization of tissues was performed, and mononuclear cells were obtained subsequently by Lympholyte M (Accurate Chemical & Scientific Corporation, Westbury, NY) density gradient centrifugation and yielded >95% viability, as determined by trypan blue exclusion. Mucosal effector lymphocytes were isolated, similar to that previously described, using collagenase digestion methods [25], [27] and also yielded >95% viability, as determined by trypan blue exclusion. Lymphocytes were incubated on OVA-coated, mixed cellulose ester membrane-bottom microtiter plates (MultiScreen-HA; Millipore, Bedford, MA). For total IgA or IgG AFC responses, wells were coated with 5 µg/ml goat anti-mouse IgA or IgG Abs (Southern Biotechnology Associates) in sterile PBS. Following overnight incubation at 37°C and a wash step, HRP-labeled anti-IgG or anti-IgA Abs were added for overnight incubation at 4°C in a humidified chamber. Following a wash step, individual wells were developed by adding 3-amino-9-ethylcarbazole substrate (AEC; Moss), and the reaction was stopped with H_2_O. Wells were allowed to dry overnight, and AFCs were enumerated, using a Stereozoom 5 dissecting microscope (Leica, Buffalo, NY).

### Cytokine detection assays

Splenic lymphocytes were isolated, as described above, by Lympholyte-M (Accurate Chemical & Scientific Corporation) gradient centrifugation. CD4^+^ T cells were isolated by negative selection as described above. An aliquot of 2×10^6^ CD4^+^ T cells was cultured for 72 h (37°C, 5% CO_2_) with an equal number of splenic feeder cells (T cell-depleted, mitomycin C-treated) in the presence or absence of 1 mg/ml OVA [25], [26]. Stimulated lymphocytes were evaluated by IFN-γ- and IL-17A-specific ELISA [25], [27], [28] and IL-4-, IL-6-, IL-10-, and IL-13-specific ELISPOT assays to enumerate cytokine-forming cell (CFC) responses, as described previously [25].

### Statistical analysis

The two-way ANOVA (Holm-Sidak method) test was used to evaluate the differences among groups in dose-dependent experiments. The one-way ANOVA test was used to evaluate the differences among experimental parameters in each experiment.

## Supporting Information

Figure S1 **Dose and kinetic analysis of nasal OVA immunization to stimulate immune Abs.** Groups of C57BL/6 mice (5–8/group) were immunized nasally with 30 µg OVA alone, 100 µg OVA alone, 30 µg OVA plus cholera toxin (CT), or 100 µg OVA plus CT using the vaccination schedule described in Fig. 4. **A.** serum IgG, **B.** serum IgA, **C.** fecal IgA, and **D.** day 35 nasal wash IgG and IgA endpoint anti-OVA Ab titers were determined by OVA-specific ELISAs. It was found that 100 µg OVA plus CT induced optimal mucosal IgA responses; *P≤0.001, **P≤0.012, *** *P*<0.05 vs. 30 µg OVA-immunized mice; and ^‡^ *P*≤0.013 vs. 100 µg OVA-immunized mice.
(TIF)

Figure S2 **IgG subclass anti-OVA responses by mice immunized with OVA plus CT.** The OVA plus CT-immunized mice in Figure S1 were evaluated for their **A.** IgG1, **B.** IgG2a, and **C.** IgG2b subclass responses to OVA on days 21 and 28 post-primary vaccination as mean ± SEM. There were no statistical differences between immunization groups measured at day 21 or 28.
(TIF)
