# Supplementary figures and images for: Loss of Sialic Acid Binding Domain Redirects Protein σ1 to Enhance M Cell-Directed Vaccination
Source: PLoS One. 2012 Apr 30;7(4):e36182. doi: 10.1371/journal.pone.0036182 (PMC3340367; doi:10.1371/journal.pone.0036182)

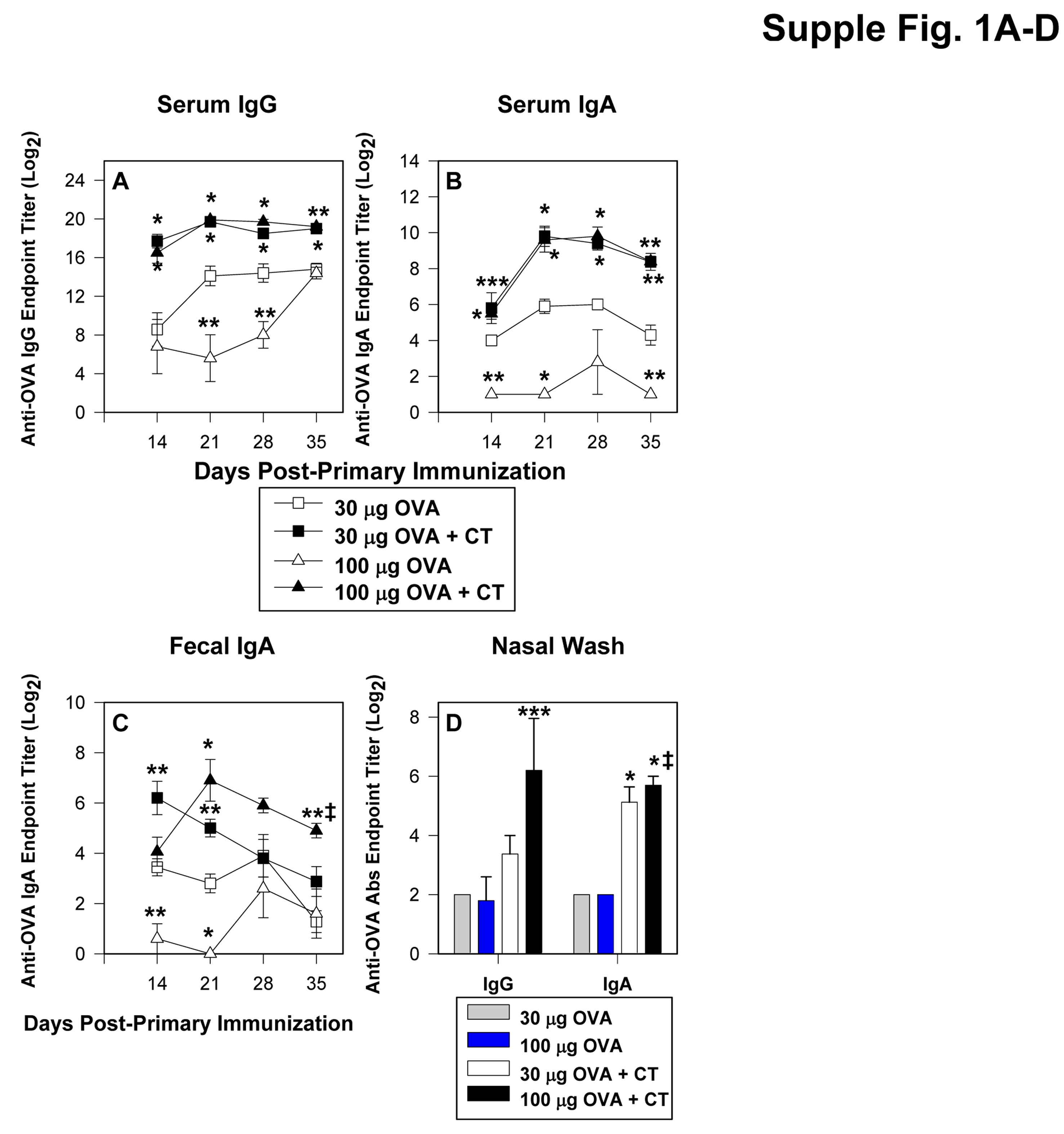

Supplement: Figure S1 — Dose and kinetic analysis of nasal OVA immunization to stimulate immune Abs. Groups of C57BL/6 mice (5–8/group) were immunized nasally with 30 µg OVA alone, 100 µg OVA alone, 30 µg OVA plus cholera toxin (CT), or 100 µg OVA plus CT using the vaccination schedule described in Fig. 4. A. serum IgG, B. serum IgA, C. fecal IgA, and D. day 35 nasal wash IgG and IgA endpoint anti-OVA Ab titers were determined by OVA-specific ELISAs. It was found that 100 µg OVA plus CT induced optimal mucosal IgA responses; *P≤0.001, **P≤0.012, *** P<0.05 vs. 30 µg OVA-immunized mice; and ‡ P≤0.013 vs. 100 µg OVA-immunized mice. (TIF) [file pone.0036182.s001.tif]

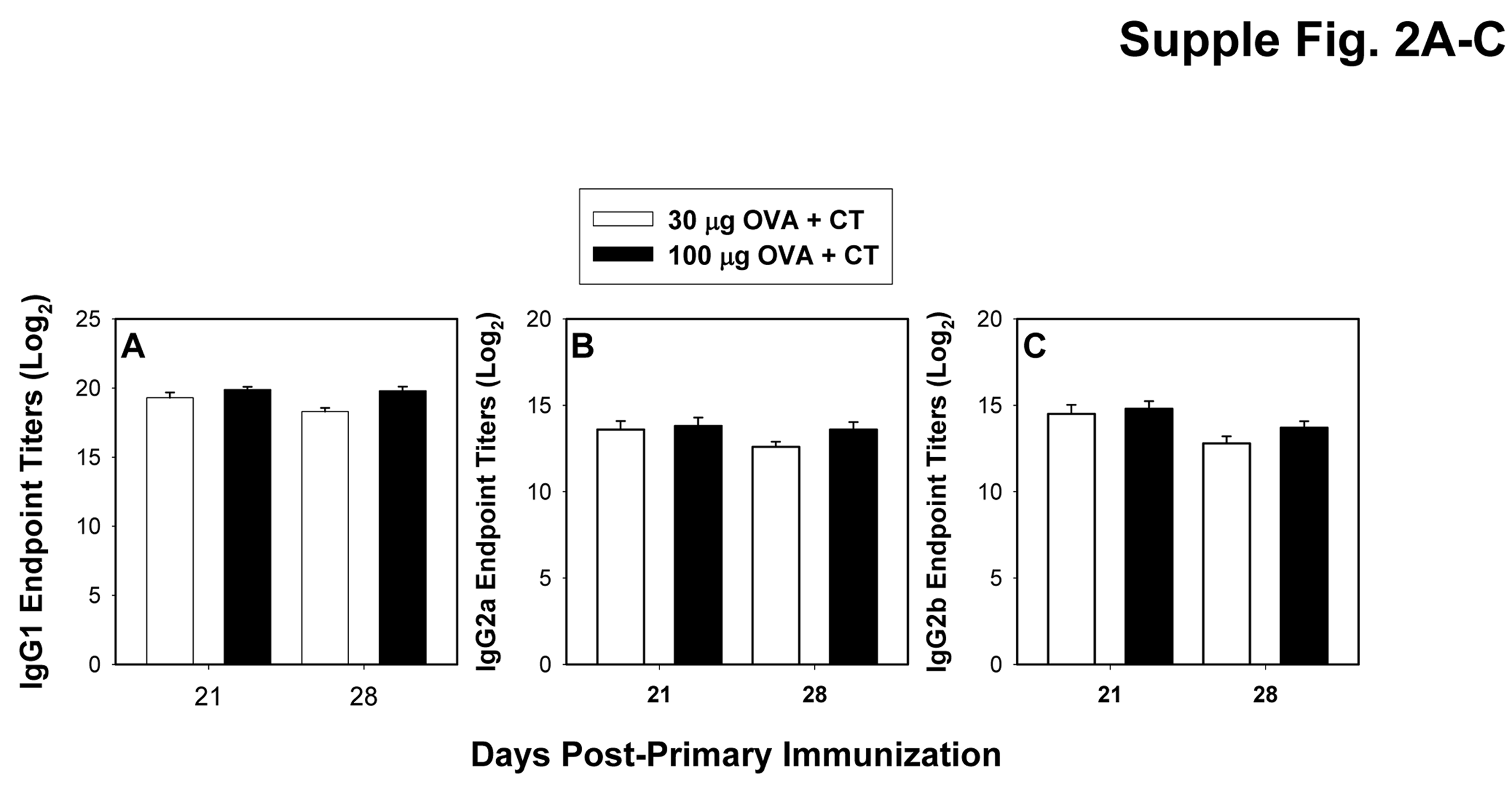

Supplement: Figure S2 — IgG subclass anti-OVA responses by mice immunized with OVA plus CT. The OVA plus CT-immunized mice in Figure S1 were evaluated for their A. IgG1, B. IgG2a, and C. IgG2b subclass responses to OVA on days 21 and 28 post-primary vaccination as mean ± SEM. There were no statistical differences between immunization groups measured at day 21 or 28. (TIF) [file pone.0036182.s002.tif]
